# Rapid and Sensitive Quantification of Nano- and Microplastics in Water, Sediment, and Biological Tissue by Pyrolysis-Gas Chromatography Tandem Mass Spectrometry with Dynamic Reaction Monitoring

**DOI:** 10.1021/acs.analchem.5c05604

**Published:** 2025-12-17

**Authors:** M. Bryan Gahn, Marcus Wharton, Asif Mortuza, David Hala, Christopher D. Marshall, Karl Kaiser

**Affiliations:** † Department of Marine Biology, 14736 Texas A&M University, Galveston, Texas 77553, United States; ‡ Department of Marine and Coastal Environmental Sciences, 33604 Texas A&M University, Galveston, Texas 77553, United States; § Department of Ecology and Conservation Biology, 171801 Texas A&M University, College Station, Texas 77843, United States; ∥ Department of Oceanography, Texas A&M University, College Station, Texas 77843, United States

## Abstract

A highly sensitive and selective method was developed for the quantification of nano- and microplastics (NMPs) in water, sediments, and biological tissues using pyrolysis gas chromatography coupled with triple quadrupole mass spectrometry (Py-GC-qQq-MS). Dynamic multiple reaction monitoring (DMRM) and internal standard calibration enabled quantification of 12 common polymers at nanogram levels (1–126 ng). Sample preparation and cleanup was matrix-specific, employing filtration, enzymatic digestion, or pressurized liquid extraction. The addition of calcium carbonate (CaCO_3_) enhanced signal intensity for several polymers, while tandem mass spectrometry ensured high specificity and sensitivity. Lipid interference for the major plastics polyethylene, polypropylene and nylon-66 was addressed by developing a rigorous correction procedure. The method was applied to field samples from the Gulf of Mexico and the Texas coast, successfully detecting NMPs in water, sediment, and biota. The presented method enables high-throughput, targeted monitoring of plastic pollution across diverse environmental matrices.

## Graphical abstract

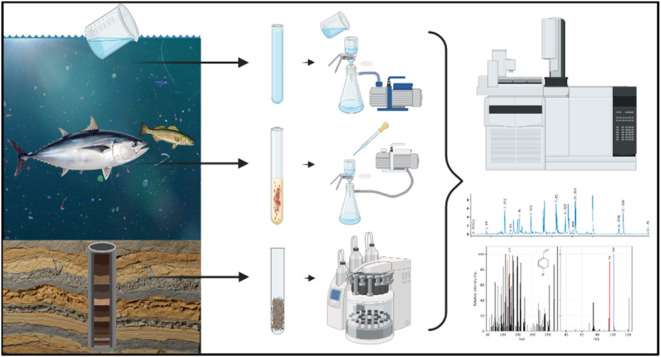

## Introduction

Global plastic production has surged over the past half-century, rising from approximately 1.3 Mt in 1950 to approximately 359 Mt in 2018.^1^ This increase has led to a growing concern regarding plastic pollution, particularly nano and microplastics (NMPs). Microplastics are generally defined as plastic particles with a maximum dimension of ≤ 5 mm, while nanoplastics typically range from 1 to 1000 nm in size.^2^ NMPs encompass a wide variety of polymer particles that originate either from direct manufacture (e.g., microbeads and synthetic textiles) or from environmental degradation processes (e.g., photooxidation and mechanical abrasion) of larger plastic debris (i.e., macroplastics, >5 mm).^3^

NMPs are ubiquitous in the environment. They have been detected from Antarctic coast sediments to Arctic ice sheets, the summit of Mt. Everest to the depths of the Mariana Trench, and have been found in organisms across all trophic levels, including humans.^4–10^ The pervasive presence of NMPs has raised significant ecological and human health concerns. NMPs can act as vectors for environmental pollutants. Hydrophobic organic contaminants (e.g., PCBs and PAHs) readily adsorb onto their surfaces, and plastics additives such as phthalates and flame retardants can leach into surrounding media (e.g., water, sediment, tissue).^11^ Even without associated harmful chemicals, the physical presence of NMPs can lead to increased production of reactive oxygen species in cells, resulting in an inflammatory response.^12^ Given the widespread NMP pollution and its complex ecological ramifications, there is a critical need for more robust high-throughput quantitative methods to accurately assess NMPs in diverse matrices.

Numerous analytical techniques have been employed for the detection and quantification of NMPs, which often combine traditional microscopy with advanced spectroscopic and molecular approaches.^13^ These methods yield either estimates of particle counts or mass-based concentrations normalized to sample volume or weight. Prior to analysis, most methods require extensive chemical or enzymatic digestion and subsequent cleanup steps to isolate plastic particles and reduce matrix complexity.^14,15^ Particle count-based methods encompass optical microscopy, laser diffraction particle size analysis, scanning electron microscopy, flow cytometry, and Fourier-transform infrared (FTIR) and Raman spectroscopy.^16^ These methods provide quantitative estimates of particle abundance across the entire size spectrum of NMPs, and some can also differentiate between plastic types.

Thermal decomposition coupled with gas chromatography–mass spectrometry (GC-MS) is an increasingly utilized technique for the analysis of NMPs.^17,18^ Pyrolysis-based methods thermally decompose plastic polymers in samples under oxygen-free conditions at temperatures exceeding 500 °C generating characteristic volatile fragments. These fragments are then separated by gas chromatography and detected by mass spectrometry with high sensitivity and specificity. As with other analytical techniques, pyrolysis-GC-MS (PyGC-MS) performance can be improved by incorporating sample cleanup steps prior to analysis to remove interfering organic and inorganic constituents. Matrix interference can often lead to misidentification of plastic polymer types and disrupt the pyrolysis process, ultimately compromising analytical accuracy and precision.^19^ For example, lipids in biological tissues can yield similar combustion products to those of polyethylene (PE), polypropylene (PP), and nylon-66 (N-66).^20^

Improvements that enable more reliable quantification of NMPs at ultralow concentrations by PyGC-MS include optimized sample treatment methodologies that include the addition of reactants to improve pyrolysis efficiency of plastic polymers, and the application of advanced mass spectrometric methods. Quantification of NMPs at trace levels is often constrained by the minimum weight limits of analytical balances, as calibration relies on the mass of particulate standards. This limitation has been addressed by using inert fillers, such as calcium carbonate (CaCO_3_) or clean quartz sand that are mixed with plastic standards to produce weighable calibration standards down to the nanogram level. While single-quadrupole mass detectors are widely used due to their simplicity and low cost, they offer limited sensitivity and are less sensitive in complex matrices.^21^

The application of time-of-flight and orbitrap mass spectrometry, as well as modern triple-quadrupole mass detectors, promises improved sensitivity and specificity of NMP quantification in complex matrices. These highly selective detectors enable precise mass determination, reducing ambiguity in peak identification and enhancing the ability to differentiate plastics from natural organic matter commonly present in environmental samples. Time of flight and orbitrap mass spectrometers support untargeted screening and identification of emerging or unknown plastic-related compounds, while triple-quadrupole instruments excel in targeted quantification with high throughput and reproducibility, which are advantageous features for routine monitoring.^22–24^ Compared to traditional methods such as FTIR or Raman spectroscopy, modern mass spectrometric approaches are better equipped to address the challenges posed by complex sample matrices, diverse plastic compositions, and trace-level concentrations, making them essential tools for future NMP analysis.^25,26^

Here we present a novel analytical method for the identification and quantification of NMPs in diverse environmental matrices that include marine waters, sediments, and biological tissues by using pyrolysis gas chromatography coupled with triple quadrupole mass spectrometry (PyGC-qQq-MS). Depending on the sample matrix, NMPs were concentrated through filtration, extraction, or digestion prior to analysis. Gas chromatography with qQq-MS detection using dynamic multiple reaction monitoring (DMRM) mode with an internal standard yielded significantly improved selectivity and sensitivity, enabling accurate and precise quantification of plastic polymers. Method validation demonstrated the robust performance of the method across diverse sample types, with reliable detection of NMPs at amounts of 1–126 ng and in the presence of significant matrix interference.

## Experimental Section

### Materials

Critical components include a Teflon pump head (Jabsco, Washington, DC, USA), filter housings and 10-in. stainless-steel filter cartridges (SS316, 5 μm; Critical Process Filtration, Nashua, New Hampshire, USA). Glass filters with nominal pore sizes of 0.7 μm (GF/F) and 2.8 μm (GF/D) (Cytivia Whatman, Marlborough, Massachusettts, USA). All GFs were furnaced at 450 °C for 5 h before use. Additional components include tris­(hydroxymethyl)­aminomethane (Tris) hydrochloride solution pH 8.0 (1 mol L^–1^) and porcine pancreatic enzyme (Pez) (MilliporeSigma, Burlington, Massachusetts, USA). Toluene (HPLC grade), methanol, acetone, and CaCO_3_ (ACS grade) (J.T. Baker, Baker City, Oregon, USA). Internal standard, poly-4-fluorostyrene (PFS) (PSS, Mainz, Germany), plastic calibration standard kit containing PMMA, PP, PVC, PC, N-6, N-66, PE, PET, SBR, PU, ABS, and PS (Frontier Laboratories, Koriyama, Fukushima, Japan), along with 80 μL Eco Cup LF pyrolysis cups. Ultrapure water (18.2 MΩ·cm) was produced using a Milli-Q IQ-7000 Ultrapure Water Purification System (Merck KGaA, Germany). And last, glass microvolume syringes (SGE Analytical Science, Ringwood, Victoria, Australia) and quartz wool (Thermo Fisher Scientific, Waltham, Massachusetts, USA) were also used.

### Surface Water Collection and Concentration

Surface water samples were collected at multiple stations along a salinity gradient in Galveston Bay, Texas. Stainless steel (SS) filter cartridges were precleaned by sonication and rinsing with ultrapure water to remove any particulates prior to sampling. Large volume (50–100 L) of surface water samples were collected using a pump filtration system capturing particulates <5 mm and >5 μm (Figure 1). Filtered sample volume was determined using a flow meter positioned downstream of the filter cartridge. Following filtration, SS filters were kept in the housing and transported to the laboratory. Housings with SS filters were sonicated and rinsed three times with ultrapure water. All rinse fractions were pooled and vacuum filtered through 47 mm GF/F filters using a glass filtration system (Figure 1). The filters were subsequently dried at 35 °C and stored for analysis.

**Figure 1:**
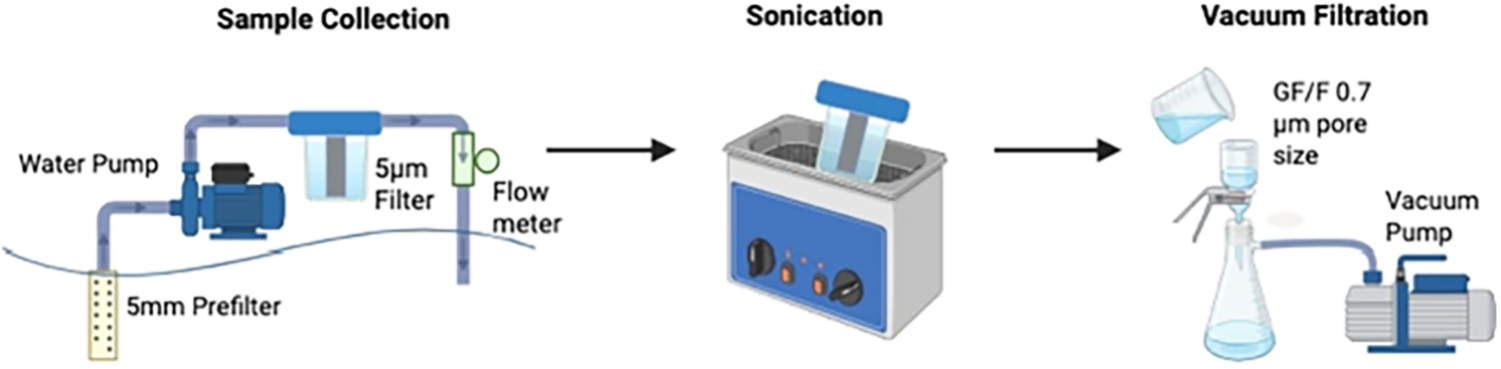
Water Collection Methodology Workflow. Surface water pump and filtration assembly for sample collection, sonication, and vacuum filtration on 0.7 μm GF/F filters.

### Sediment Collection and Concentration

Sediment samples collected from various locations and environments along the Texas coast were dried and homogenized in a SPEX SamplePrep 8000 M Mixer/Mill for 5 min. Plastics were extracted using a pressurized liquid extraction (PLE) method with a Dionex ASE 350 system.^27^ Approximately 5 g of each homogenized sample, mixed with sufficient clean Ottawa sand to fill the void space, was loaded into precleaned 34 mL ASE cells for PLE. An internal surrogate standard PFS was added to each cell before extraction. Extraction was performed using dichloromethane (DCM) at 180 °C and 1500 psi, with a heating time of 5 min and static time of 5 min for each of the three extraction cycles. Detailed extraction parameters are listed in Table S1. Extracts were dried under N_2_ and resuspended in dichloromethane before being transferred to pyrolysis cups for analysis.

### Enzymatic Digestion of Biological Tissues

NMPs present in biological tissues were extracted by enzymatic digestion (Figure 2) using a modified method based on von Friesen et al.^28^ An enzyme solution was prepared by dissolving 6 g of Pez, which includes protease, amylase, and lipase, in 100 mL of 1 mol L^–1^ Tris hydrochloride solution (pH 8.0), yielding a final concentration of 0.06 g mL^–1^. The Pez/Tris solution was heated at 40 °C for 3 h and subsequently filtered through GF/D and GF/F to reduce background NMP contamination introduced by the enzyme.

**Figure 2:**
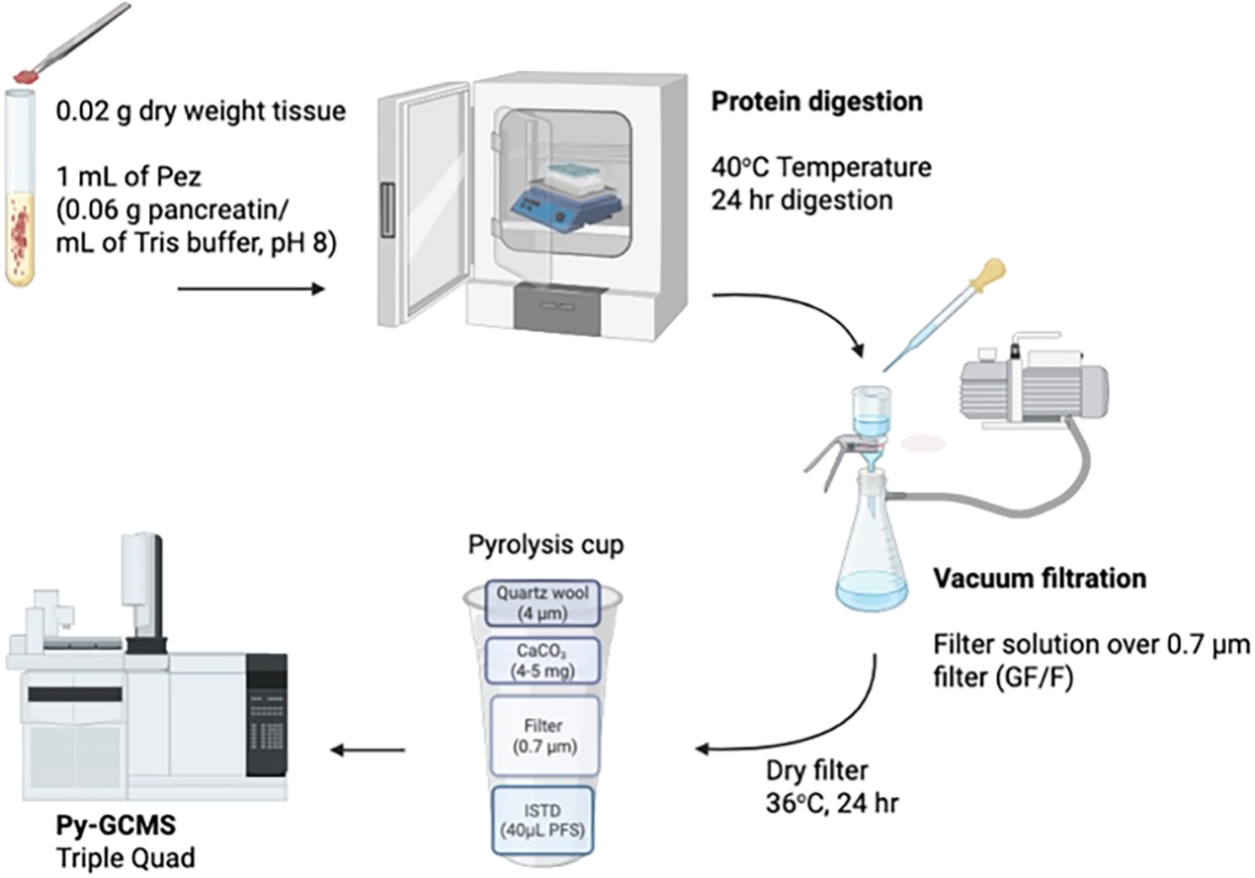
Enzymatic digestion protocol of tissue samples. Muscle samples were treated with pancreatin and Tris buffer, placed in a shaker tray and oven overnight, vacuum filtered through a GF/F filter, and dried overnight. Dried filter in an eco-cup LF with internal standard, calcium carbonate and quartz wool, then ran via PyGC-qQq-MS.

Approximately 20 mg of freeze-dried biological tissue samples (fish or oyster tissue) were placed into round-bottom glass vials and treated with 1 mL of the Pez/Tris solution. The samples were incubated for 24 h at 38–40 °C with continuous shaking on a shaker tray, maintaining an optimal pH of 8 for enzyme activity.^29^ Following digestion, samples were vacuum filtered through 25 mm GF/F. Filters were rinsed with ultrapure water, and the unused filter area was removed with a cork borer. The remaining section was dried for 24 h at 38 °C in covered glass Petri dish.

### Lipid Extraction

Freeze-dried and ground tissue (∼0.5 g), along with sufficient clean Ottawa sand (standard 20–30 mesh, Spectrum Chemical) to fill the void space, was subjected to pressurized liquid extraction in precleaned 34 mL ASE cells using an ASE 350 system (Dionex, Sunnyvale, CA). Extraction was performed using a 1:1 mixture of dichloromethane and hexane at 100 °C and 1500 psi. Each extraction included a 5 min preheating phase and a separate 5 min heating phase, followed by a 4 min static period. This cycle was repeated twice. The rinse volume was 40%, and the purge time was set to 300 s. Detailed extraction parameters are listed in Table S2. The resulting solvent extracts were collected in 50 mL amber glass bottles and dried under a gentle stream of N_2_.

### Pyrolysis GC/MS qQq-MS Analysis

Pyrolysis gas chromatography was carried out with a Frontier Auto-Shot AS-2020E sampler and an EGA/PY-3030D multishot pyrolyzer connected to an Agilent 8890 GC and 7010B qQq-MS. Prior to pyrolysis at 600 °C, GF/F with particulates from water samples or digested tissues were spiked with PFS, which served as an internal standard. Filters were then transferred to Frontier Eco Cups and topped with 5 mg of CaCO_3_ and quartz wool to ensure proper packing. Redissolved sediment extracts were transferred directly into the Eco Cup LFs, dried under a gentle stream of N_2_, and packed with CaCO_3_ and quartz wool.

The split ratio was set to 50:1, with a column flow of 0.8 mL min^–1^ using helium as the carrier gas. The injector inlet temperature was set to 300 °C. The septum purge flow was 20 mL min^–1^. Straight liners packed with nondeactivated glass wool were used. Separation was achieved on a Frontier Ultra Alloy 5 (UA5-30M-0.25F) column with the following temperature program: start temperature 35 °C, hold for 0.25 min, ramp at 20 °C min^–1^ to 310 °C, hold for 3 min, totaling runtime at 17 min. The MS detector settings were as follows: ion source temperature 230 °C, quadrupole temperature 150 °C, and auxiliary temperature 280 °C, with a 5 min solvent delay. Helium at 2.25 mL min^–1^ was used as the quench gas, and nitrogen at 1.5 mL min^–1^ served as the collision gas. Collision energy was 20 eV for all transitions. Cycle time for DMRM acquisitions was 2.5 cycles s^–1^. The detector was regularly autotuned using routines provided by the Agilent ChemStation software. The quantification of major plastic types was based on selected reaction monitoring (SRM) using characteristic product ions listed in Table 1. Qualifier ions were monitored parallel to the quantifier ions to confirm plastic analyte identity. Both quantifier and qualifier ions were selected by analyzing plastic standards and evaluating background ions from the organic matter matrices present in biological particulates in full scan mode (50–650 *m*/*z*). Quantification was based on internal standard calibration using PFS as the internal standard. Calibration standards consisted of homogeneous mixtures of plastics in CaCO_3_ to enable calibration at ng and μg levels.

**Table 1:** Plastic Polymer Abbreviations, Relative Retention Times (RRT), Characteristic Ions, and Limit of Detection (LOD)

Plastic type	Abbreviation	RRT^a^	Precursor (*m*/*z*)	Product ions^b^ (*m*/*z*)	LOD^c^ (ng)
Poly(methyl methacrylate)	PMMA	0.519	100	*41*, 39	31
Polypropylene	PP	0.591	126	*55*, 41	12
Polyvinyl chloride	PVC	0.677	128	*102*, 76	41
Nylon-6	PA	0.731	113	*56*, 30	1
Polycarbonate	PC	0.787	134	119, *91*	10
Nylon-66	N-66	0.796	84	55, *41*, 39	30
Polyethylene	PE	0.857	82	*67*, 41	60
Polyethylene Terephthalate	PET	0.955	182	*105*, 77	7
Poly(4-fluorostyrene)	PFS*	1.000	122	101, *96*	126
Polyurethane	PUR	1.206	198	*93*, 77	1
Acrylonitrile butadiene styrene	ABS	1.215	170	143, *128*	4
Styrene butadiene rubber	SBR	1.248	212	*170*, 119	104
Polystyrene	PS	1.324	312	129, *91*	1

a *Retention times are relative to PFS as internal standard on a Frontier Ultra Alloy 5 column.

b Product ions are used for SRM quantification, italicized ions are used for quantification, and other ions were used as qualifiers.

c LOD was calculated using S/*N* = 3.

## Results and Discussion

### Pyrograms

The method was optimized to yield characteristic pyrolytic products of plastic polymers that were well resolved on a Frontier Ultra Alloy 5 column with a linear temperature program. Pyrograms of surface water, vertebrate and invertebrate tissue, and sediment samples, alongside a standard plastic mixture are shown in Figure 3. Pyrolysis temperature was optimized to ensure efficient volatilization of polymers, and specific product ions were chosen to minimize matrix influence that can generate pyrolysis products with similar mass spectra. For each polymer, precursor and product ions were selected to minimize interference from natural polymers and characteristic fragmentation reactions. For polymers that primarily undergo depolymerization (e.g., PE, PP, PS, PMMA), the selected precursor ions corresponded to characteristic oligomeric or monomeric pyrolysis products, and the quantifier ions were derived from simple bond cleavages or rearrangement reactions. For example, PS pyrolysis products included mono, di-, and trimeric units with different retention times. Mono- and dimeric fragments had retention times and mass spectra that overlapped with matrix peaks of similar mass ions, whereas the trimeric PS fragment eluted without interference from matrix components.

**Figure 3:**
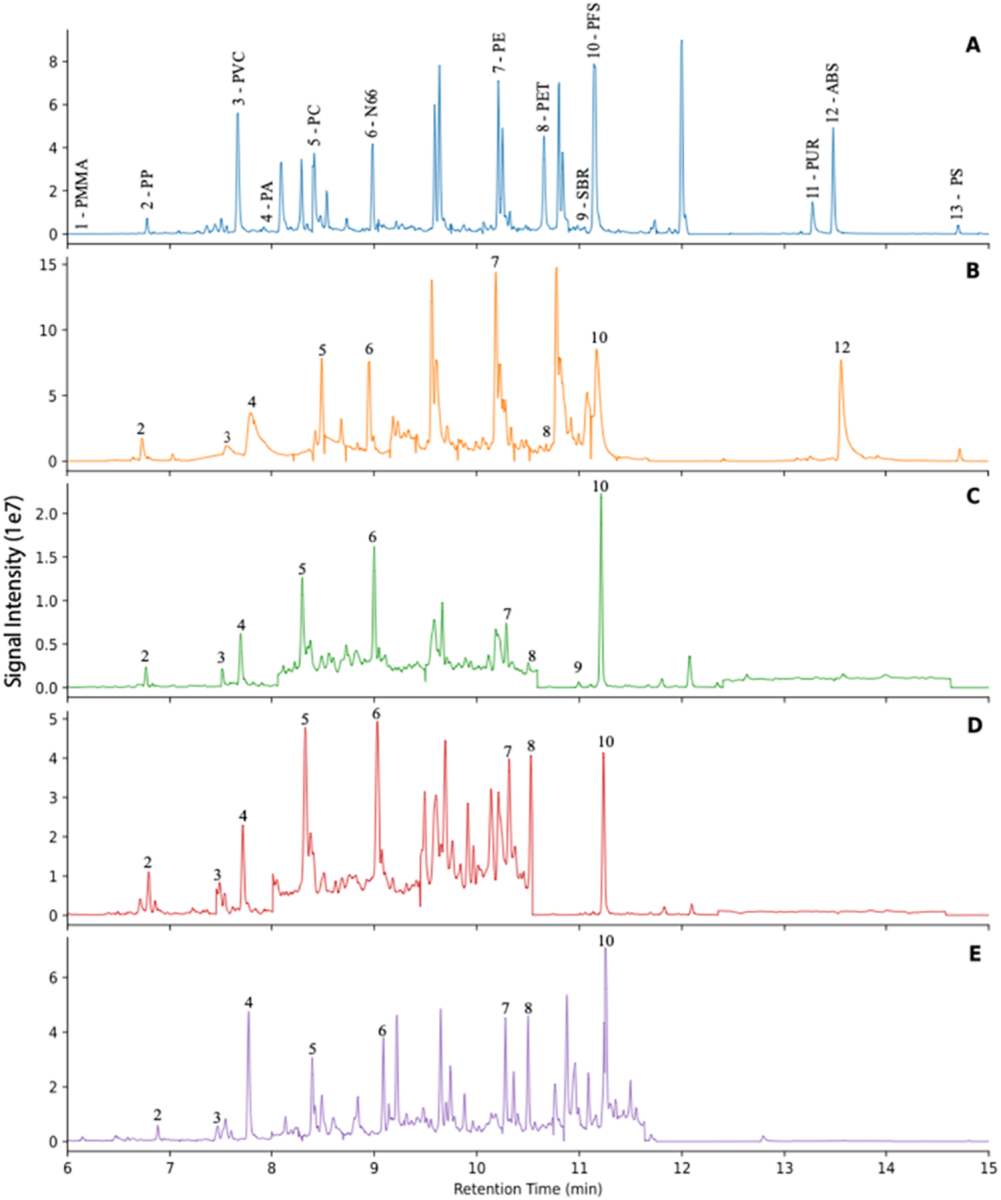
Pyrograms of (A) Calibration standard composed of homogenized mixture containing 12 plastics of interest; (B) Surface water samples collected in Galveston Bay (2019); (C) Mullet muscle tissue collected in Galveston Bay (2021); (D) Oyster tissue collected in Galveston Bay (2021); (E) Benthic sediment collected from Matagorda Bay (2023).

CaCO_3_ was added to standards and samples to act as a catalyst during pyrolysis, which lowered the LOD for all polymers except PS and PMMA. In the presence of CaCO_3_, peak intensities of N-66 and PA increased by 5-fold and 2-fold, respectively. For PC, PUR, PET, PA, and N66 the presence of CaCO_3_ promoted the conversion of reactive pyrolysis products into more GC-amenable products such as 4-isopropenylphenol, 4,4′-methylenedizniline, benzophenone, ε-caprolactam, and cyclopentanone.^30^ These characteristic products arose from interaction with CaCO_3_ rather than thermal cleavage alone, which enhanced the formation of the characteristic products. Mechanistically, ionization pathways correspond to either straightforward depolymerization to oligomeric alkenes or monomers (PE, PP, PS, PMMA), or CaCO_3_ catalyzed rearrangement and condensation of oxygen containing or nitrogen containing intermediates (PC, PUR, PET, PA, N66).

Sample processing and tissue digestion yielded clean pyrograms with minimal coelution. Residual sample matrix led to the buildup of char within the quartz tube of the pyrolysis furnace and in the interface needle, where the pyrolysis products were transferred to the GC column. The extent of charring was proportional to the amount of organic material present in the samples and affected ion abundances. Placing glass wool on top of sample cups helped reduce the transfer of char to the quartz liner and interface needle.

A rigorous cleaning protocol was implemented to maintain the proper functioning of the pyrolysis furnace and prevent gas leaks. These leaks arose from the high combustion temperature and the periodic failure of seals and thermal expansion of components. Typically, the furnace and injection system were cleaned after every 50 samples, but more frequent cleaning was necessary if fish tissues with high lipid content (>2%) were analyzed. In parallel with furnace cleaning, the ion source of mass spectrometer was also cleaned to resolve decreasing ion abundances due to the residue build-up in the ion source.

### QqQ-MS Detection and Quantification

Optimal DMRM parameters for the detection of plastics were established using pure polymer standards (Table 1). Characteristic and abundant molecular precursor ions were identified for each plastic polymer and evaluated against natural polymers to minimize matrix interference. Fragmentation ions derived from these precursor ions were selected based on characteristic fragmentation reactions. The more abundant product ion was used for quantification, whereas the secondary ion was employed as the qualifier to monitor potential coelution with matrix compounds. Fragment losses usually corresponded to the loss of polymer subunits or dehydration reactions. Operating in DMRM mode improved sensitivity by maximizing acquisition time for each targeted analyte. Ratios of quantifier to qualifier ion (Table S2), together with retention times, ensured both compound identification and confirmation.

Quantification of plastics was based on internal standard calibration using PFS as the internal standard. PFS was dissolved in toluene and added to the sample prior to pyrolysis. The use of deuterated polystyrene as an internal standard was explored; however, extensive hydrogen–deuterium exchange occurred during pyrolysis, leading to pyrolysis products with variable D/H content.^31^ Similarly, attempts to use brominated polystyrene were unsuccessful, as bromine was readily lost from the polymer chain during pyrolysis, resulting in products indistinguishable from those of PS and others.

Calibration at <1 mg plastic mass was accomplished using plastic standards dispersed in CaCO_3_. The most consistent results were achieved with commercially available standard kits (e.g., Frontiers), which contain homogeneously distributed plastic polymers in CaCO_3_. Attempts to prepare homogeneous mixtures of plastic polymers in CaCO_3_ by ball milling finely ground plastics with CaCO_3_ yielded less consistent results than commercial standards.

### Method Validation and Performance

Reagent and procedural blanks were analyzed to ensure clean sample handling and reagent purity. These blanks typically consisted of 40 μg of internal standard (2 g L^–1^ PFS in toluene), a GF/F filter (used to filter 1 mL of Pez/Tris), ∼5 mg of CaCO_3_, and quartz wool. The enzyme pancreatin used for biological tissue digestion was found to contain measurable concentrations of plastics (N-66, PC, PE, PMMA, PA, PP, and PVC); therefore, the enzyme solution was always filtered sequentially through GF/D and GF/F filters before use. This significantly decreased total contamination in tissue blanks to 4–31 μg dependent on batches of pancreatin used. Water and sediment blanks both had <10 μg total plastic contamination.

The linearity of calibration curves for all plastics was investigated with amounts spanning over 2 orders of magnitude and ranging from 0.2 to 260 μg. Calibration ranges within 1 order of magnitude were linear, whereas calibration curves over the entire tested range were best fitted with a quadratic regression to achieve correlation coefficients (*R* ^2^) > 0.999 (Table S2). The limit of detection (LOD), calculated using a signal-to-noise ratio of 3, ranged from 2 to 126 ng across different plastic types (Table 1). The pyrolyzer and ion source were cleaned prior to this test.

Lipids in the tissues interfered with the quantification of PE, PP, and N-66 by producing similar precursor ions with identical fragmentation patterns. For accurate quantification of these plastics, a correction based on total lipid content was necessary. A correction procedure was developed by analyzing palmitic acid (PAL) and a triglyceride lipid mixture (TLM, Sigma-Aldrich and Lipid Standards: Triglyceride mixtures 17811-1AMP). PE showed the highest interference from lipids, followed by PP and N-66 (Figure 4). Lipid interference for these plastics was independent from lipid composition.

**Figure 4:**
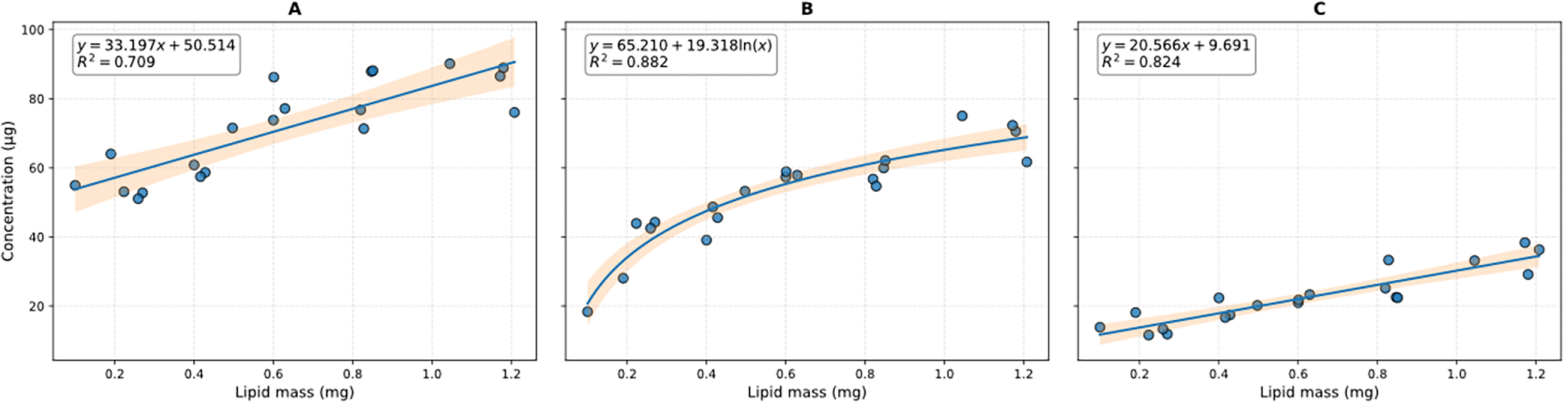
Lipid interference curves for PE (A), PP (B), and N-66 (C) with 95% confidence intervals. Measurements are based on palmitic acid and a triglyceride mixture. Relationships were used to subtract lipid interference from samples.

Precision was evaluated with replicate analyses of surface water, sediments, and fish tissues (*n* = 5, Table 2). Surface water showed consistently high precision ranging from 3 to 11% across detected polymers. Sediment analysis showed precisions ≤ 9% for all detected polymers except ABS (21%). Precision in tissues was variable and matrix dependent ranging from 23 to 200% in oysters, 8–62% in red drum, and 2–41% in spotted seatrout. Overall, the method afforded excellent precision in water and sediment matrices (generally ≤ 10% RSD) with reduced precision in biological tissues, consistent with higher matrix complexity.

**Table 2:** Replicate Measurements of Different Sample Types (*n* = 5)^a^

	RSD (%)											
Sample type	ABS	N-66	PA	PC	PE	PET	PMMA	PP	PS	PUR	PVC	SBR
Surface water	11	4	7	5	3	10	6	6	9	^b^nd	6	7
*Crassostrea virginica*	68	24	^b^nd	^b^nd	23	200	^b^nd	45	^b^nd	^b^nd	ND	75
*Sciaenops ocellatus*	^b^nd	17	^b^nd	^b^nd	32	38	^b^nd	27	8	^b^nd	34	62
*Cynoscion nebulosus*	^b^nd	26	^b^nd	38	2	20	40	12	^b^nd	^b^nd	14	41
Sediment	21	2	2	^b^nd	<1	3	^b^nd	3	9	^b^nd	2	^b^nd

a Precision is expressed as a relative standard deviation (%).

b nd = not detected.

The matrix interferences and accuracy of the method were assessed by adding known amounts of plastics to replicate samples of sediment, water, and fish tissue (*n* = 4–5). Results showed that accuracy was highest for surface water and sediment samples ranging from 73–137% and 77–101%. Accuracy in muscle tissue ranged from 94 to 111% for major plastics like N-66, PE, PP, and PVC, even with substantial lipid interference. Lower accuracies of 20–274% were observed for minor plastics such as PUR, SBR, PET and PC. The high accuracies for the dominant polymers in biological tissues demonstrated that internal standard calibration and lipid correction effectively compensated for lipid and other tissue related interferences (Table 3).

**Table 3:** Analysis of Matrix Interference and Method Accuracy^a^

	(% Recovery)		
Plastic	^b^Water	^b^Sediment	^c^Muscle
N-66	86 ± 7	98 ± 4	95 ± 17
PE	94 ± 7	101 ± 2	111 ± 11
PP	88 ± 6	92 ± 2	102 ± 5
PVC	88 ± 8	96 ± 4	94 ± 7
ABS	95 ± 7	77 ± 4	143 ± 8
PA	87 ± 4	88 ± 2	98 ± 8
PC	94 ± 17	86 ± 3	174 ± 13
PET	73 ± 13	82 ± 14	213 ± 43
PMMA	116 ± 36	90 ± 1	104 ± 7
PS	82 ± 25	74 ± 5	122 ± 26
PUR	137 ± 19	86 ± 9	20 ± 16
SBR	82 ± 6	96 ± 5	274 ± 60

a Replicate samples (*n* = 4-5) were spiked with plastic standards and recovery was quantified.

b *N* = 5; ± Standard Deviation.

c *N* = 4; ± Standard Deviation.

### Concentrations of NMPs in Surface Waters, Biological Tissues, and Sediment

The applicability of the method was further explored with surface water samples across varying salinities, biological tissues, and sediments (Figure 5). Measurements confirmed the ubiquitous occurrence of NMPs in the environment. Plastic concentrations ranged from 2 to 35 μg L**^–1^** in surface water samples, from 6.9 to 185.9 μg g^–1^ dry weight in sediments, and 0.1–13.3 mg g**^–1^** dry weight in biological tissues. Surface water was collected in Matagorda Bay and Galveston Bay (Texas, USA), in the Sabine River (Texas, USA), the Mississippi River at New Orleans (Louisiana, USA), and the Mobile River (Alabama, USA).

**Figure 5:**
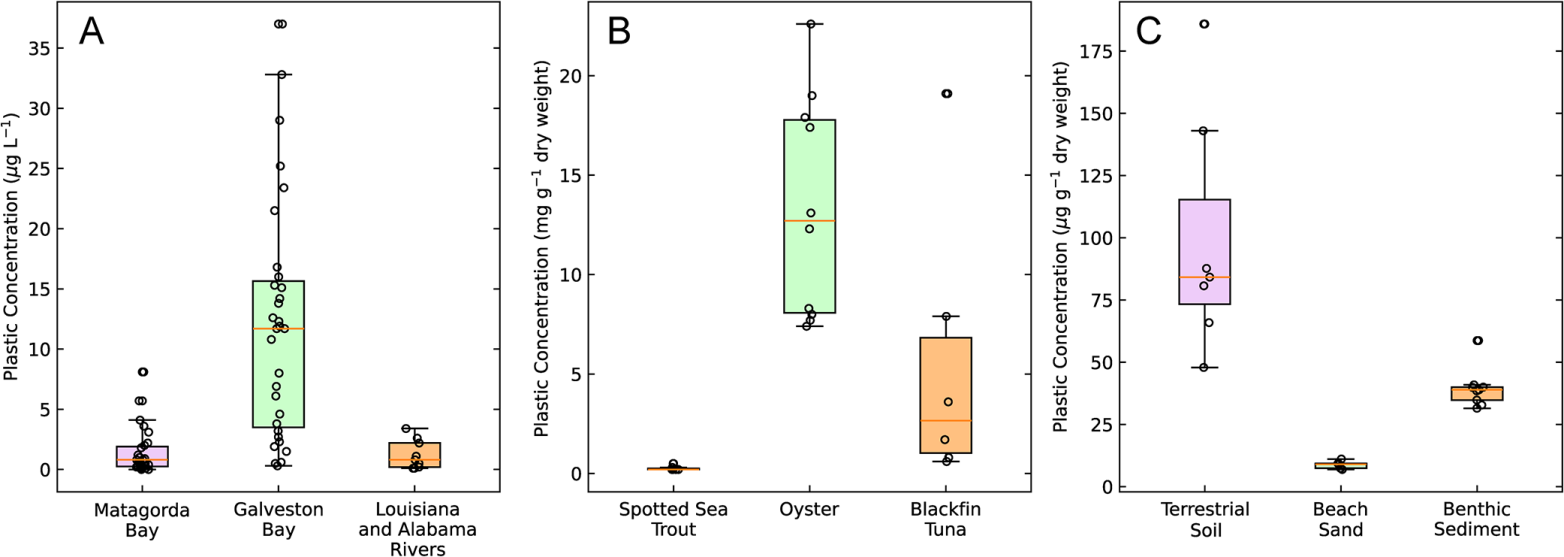
Plastic concentration in surface water samples (A), biological tissues (B) and sediment (C).

Plastic concentrations measured in Galveston Bay surface water samples were higher than those from other sites. Galveston Bay is a center of plastic production in the USA, located next to some of the world’s largest petrochemical industries and shipping ports. The Port of Houston is the largest port in the USA by total tonnage and second in terms of foreign cargo value (222.5 billion USD).^32^ The accumulation of NMPs in food webs was confirmed by high concentrations of plastics in muscle tissue of fishes and oysters (Figure 5). The spotted sea trout and oyster samples were collected from Galveston Bay (Texas, USA), while blackfin tuna were sampled from across the Gulf of Mexico. Spotted sea trout had the lowest concentrations among analyzed muscle tissues ranging from 0.2 to 0.5 mg plastic g^–1^ dry weight. Oysters are suspension feeders that can pump up to 170 L of water per g dry weight per day.^33^ Their high tissue concentrations (7.4–22.6 mg plastic g^–1^ dry weight) indicated efficient uptake and retention of NMPs within their tissues. Blackfin tuna are apex predators, and the presence of NMPs in their tissues at high concentrations (0.6–19.1 mg plastic g^–1^ dry weight) suggested potential bioaccumulation of plastics within food webs.

## Conclusion

This study developed and validated a robust analytical workflow for the detection and quantification of NMPs across a diverse range of environmental matrices. The method demonstrated high sample throughput, robust compensation of matrix interference, and high precision and accuracy. This was accomplished through efficient sample cleanup procedures, internal standard calibration, tandem mass spectrometry, and a correction for lipid interference in the detection of PE, PP, and N-66. The applicability of the method was demonstrated on marine surface waters, sediments and soils, and muscle tissue of fishes and oysters. Results revealed quantifiable concentrations of plastics in all sample types emphasizing the pervasive and persistent nature of plastics in the environment and their bioaccumulation potential across trophic levels. This method provides a critical tool for studying NMPs in the environment, ecological risks, and potential impacts on human health.

## Supplementary Material





## Notes

Fragment ion spectra for all characteristic transitions (Figures S1–S13); ASE Parameters for Sediment and Lipid Extraction (Table S1); Calibration curve *R* ^2^, residuals and qual/quant ion ratios (Table S2); and Lipid correction curve equations (Table S3) (PDF)
Raw Spectral Data (XLSX)
