# Supplementary material for: Rapid and Sensitive Quantification of Nano- and Microplastics in Water, Sediment, and Biological Tissue by Pyrolysis-Gas Chromatography Tandem Mass Spectrometry with Dynamic Reaction Monitoring
Source: Anal Chem. 2025 Dec 17;98(1):633–41. doi: 10.1021/acs.analchem.5c05604 (PMC12809636; doi:10.1021/acs.analchem.5c05604)
Supplement: Supplementary file 1 [file ac5c05604_si_001.pdf]

Supporting Information

Rapid and Sensitive Quantification of Nano- and Microplastics in Water, Sediment, and Biological Tissue by Pyrolysis-Gas Chromatography Tandem Mass Spectrometry with Dynamic Reaction Monitoring

M. Bryan Gahn[1], Marcus Wharton[2], Asif Mortuza[1], David Hala[1], Christopher D. Marshall[1,3], and Karl Kaiser[2,4*]*

[1]Department of Marine Biology, Texas A&M University, Galveston, Texas 77553, United States
[2]Department of Marine and Coastal Environmental Sciences, Texas A&M University, Galveston, Texas 77553, United States
[3]Department of Ecology and Conservation Biology, Texas A&M University, College Station, Texas 77843, United States
[4]Department of Oceanography, Texas A&M University, College Station, Texas 77843, United States

Corresponding authors:
E-mail: bryangahn@tamu.edu (M.B.G); kaiserk@tamug.edu (K.K.)

Section 1. PMMA Mass Spectra

Section 2. N66 Mass Spectra

Section 3. PA Mass Spectra

Section 4. PC Mass Spectra

Section 5. PET Mass Spectra

Section 6. PFS Mass Spectra

Section 7. PVC Mass Spectra

Section 8. PUR Mass Spectra

Section 9. PS Mass Spectra

Section 10. PE Mass Spectra

Section 11. PP Mass Spectra

Section 12. ABS Mass Spectra

Section 13. SBR Mass Spectra

Section 14. ASE Parameters

Section 15. GC-MS/MS Calibration Parameters

Section 16. Lipid Correction Equations

Section 1. PMMA Mass Spectra

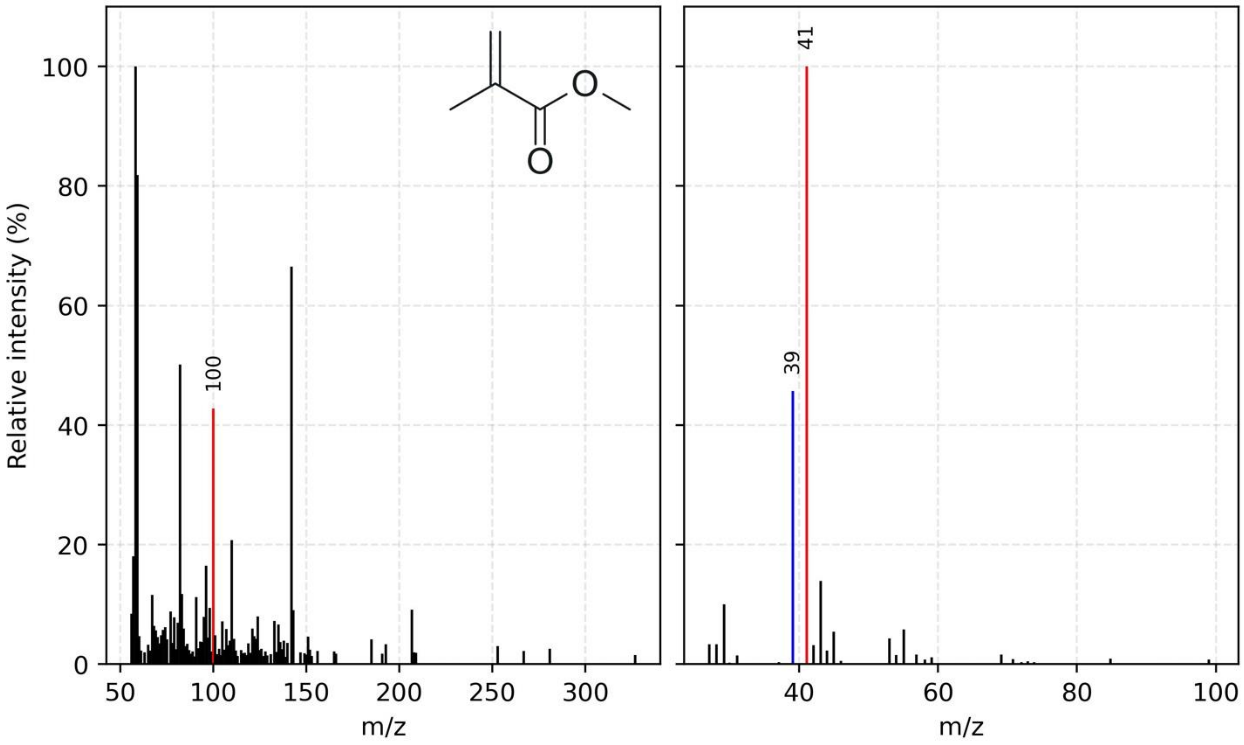

Figure S1. Full-scan (left) and product-ion (right) spectra for PMMA acquired under optimized MS/MS conditions. Quantifier (red) and qualifier (blue) ions indicate the transitions used for quantitative analysis and identity confirmation.

Section 2. N66 Mass Spectra

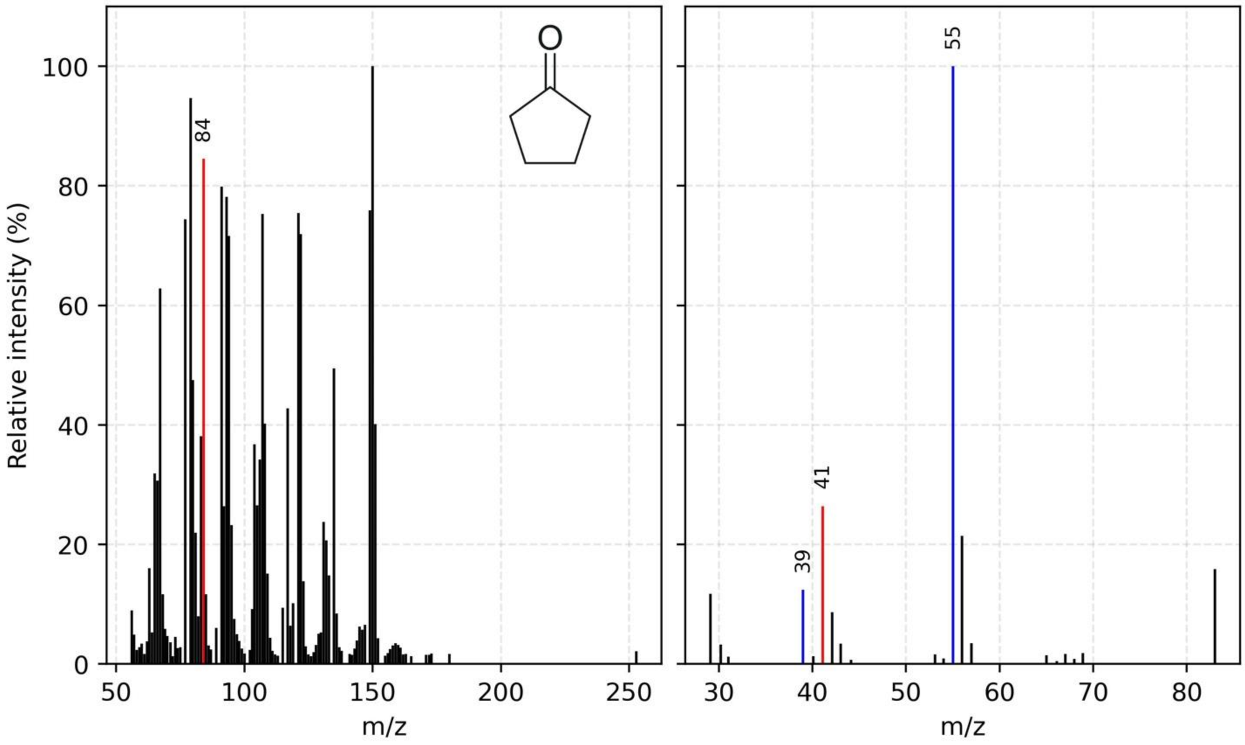

Figure S2. Full-scan (left) and product-ion (right) spectra for N66 acquired under optimized MS/MS conditions. Quantifier (red) and qualifier (blue) ions indicate the transitions used for quantitative analysis and identity confirmation.

Section 3. PA Mass Spectra

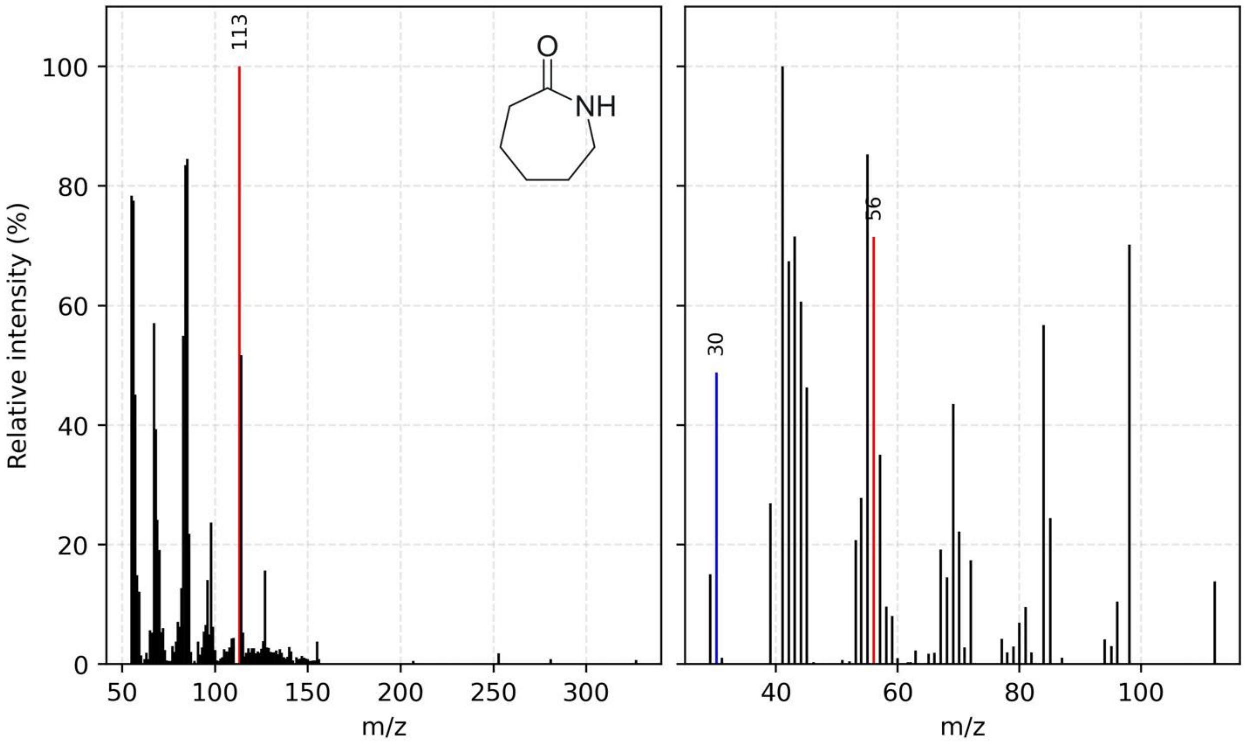

Figure S3. Full-scan (left) and product-ion (right) spectra for PA acquired under optimized MS/MS conditions. Quantifier (red) and qualifier (blue) ions indicate the transitions used for quantitative analysis and identity confirmation.

Section 4. PC Mass Spectra

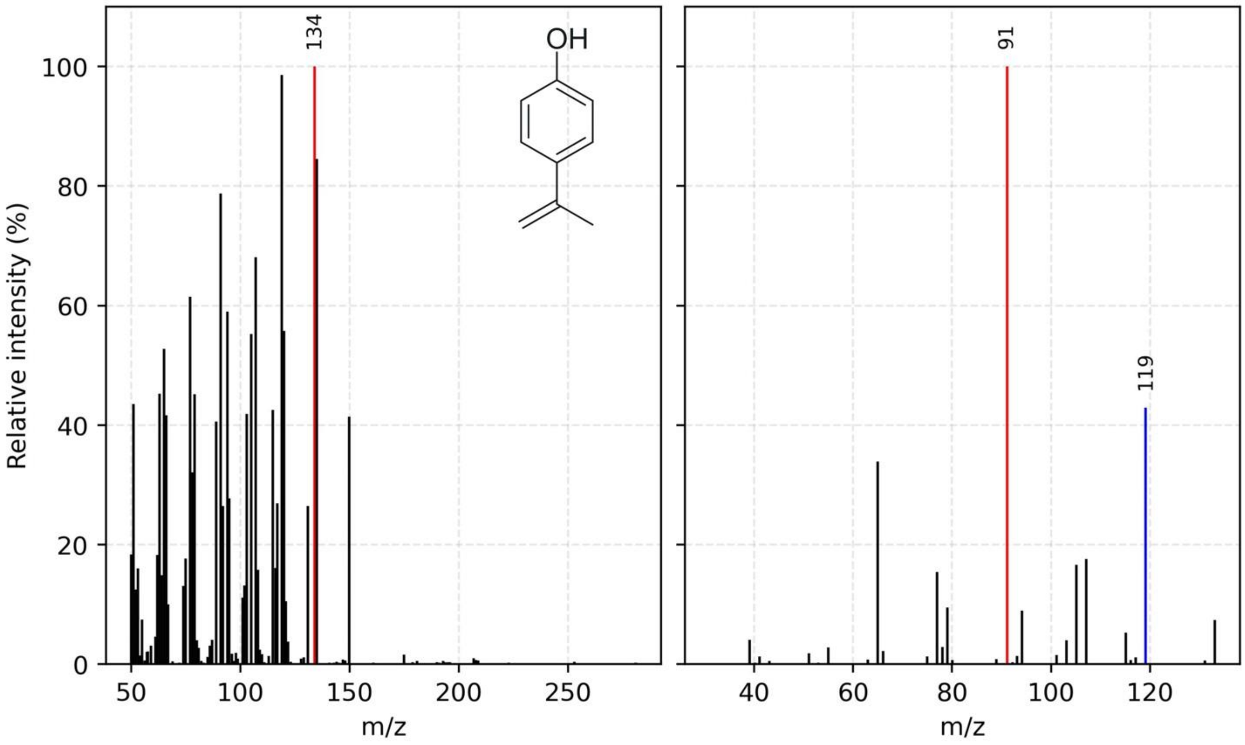

Figure S4. Full-scan (left) and product-ion (right) spectra for PC acquired under optimized MS/MS conditions. Quantifier (red) and qualifier (blue) ions indicate the transitions used for quantitative analysis and identity confirmation.

Section 5. PET Mass Spectra

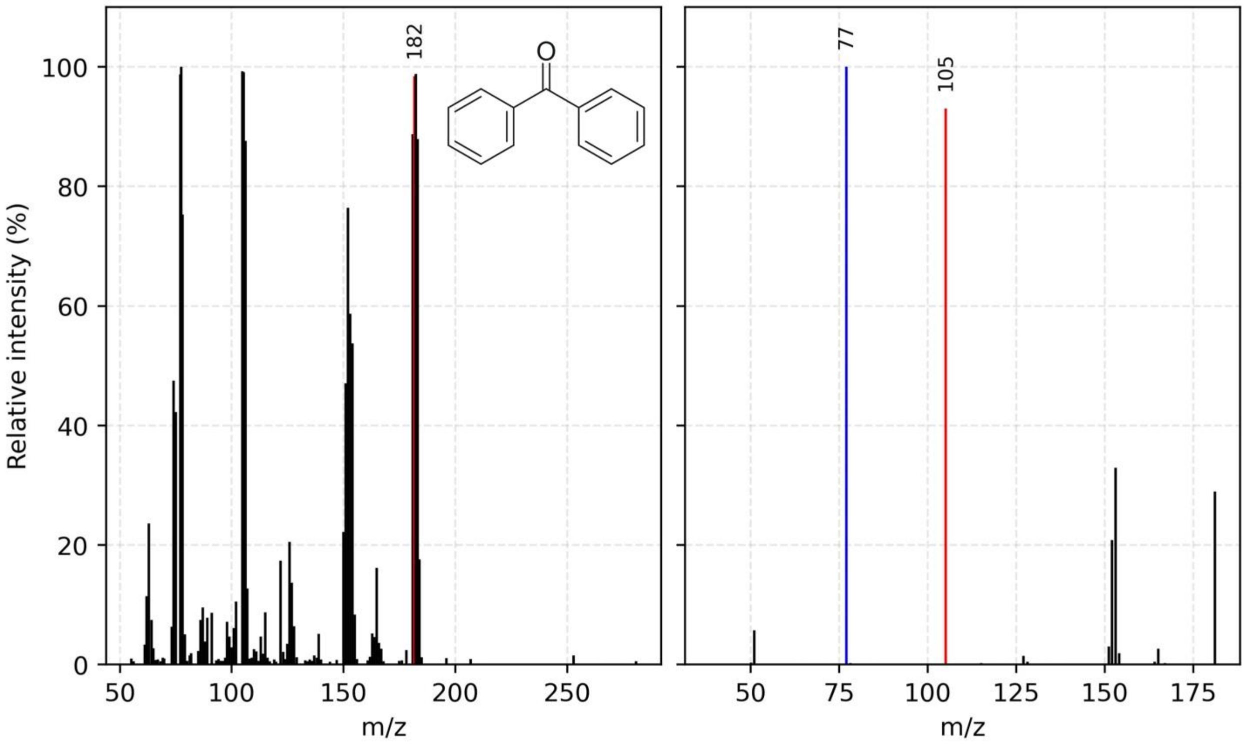

Figure S5. Full-scan (left) and product-ion (right) spectra for PET acquired under optimized MS/MS conditions. Quantifier (red) and qualifier (blue) ions indicate the transitions used for quantitative analysis and identity confirmation.

Section 6. PFS Mass Spectra

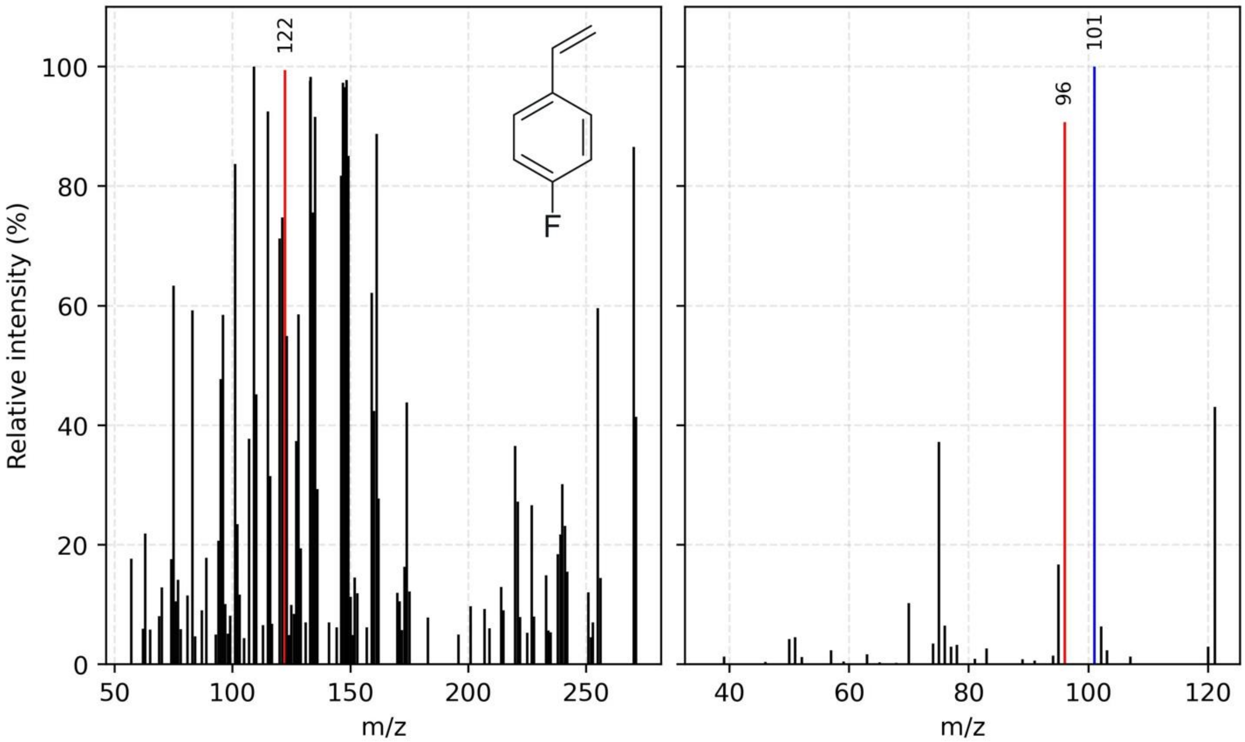

Figure S6. Full-scan (left) and product-ion (right) spectra for PFS acquired under optimized MS/MS conditions. Quantifier (red) and qualifier (blue) ions indicate the transitions used for quantitative analysis and identity confirmation.

Section 7. PVC Mass Spectra

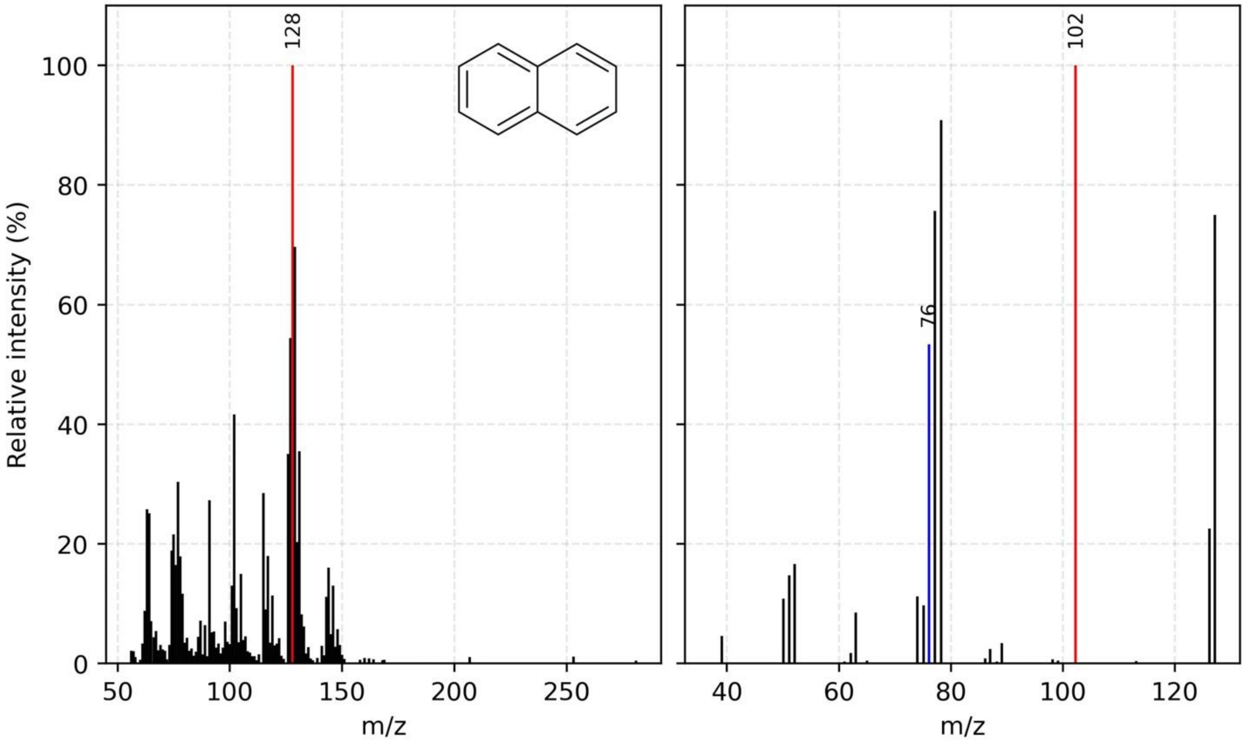

Figure S7. Full-scan (left) and product-ion (right) spectra for PVC acquired under optimized MS/MS conditions. Quantifier (red) and qualifier (blue) ions indicate the transitions used for quantitative analysis and identity confirmation.

Section 8. PUR Mass Spectra

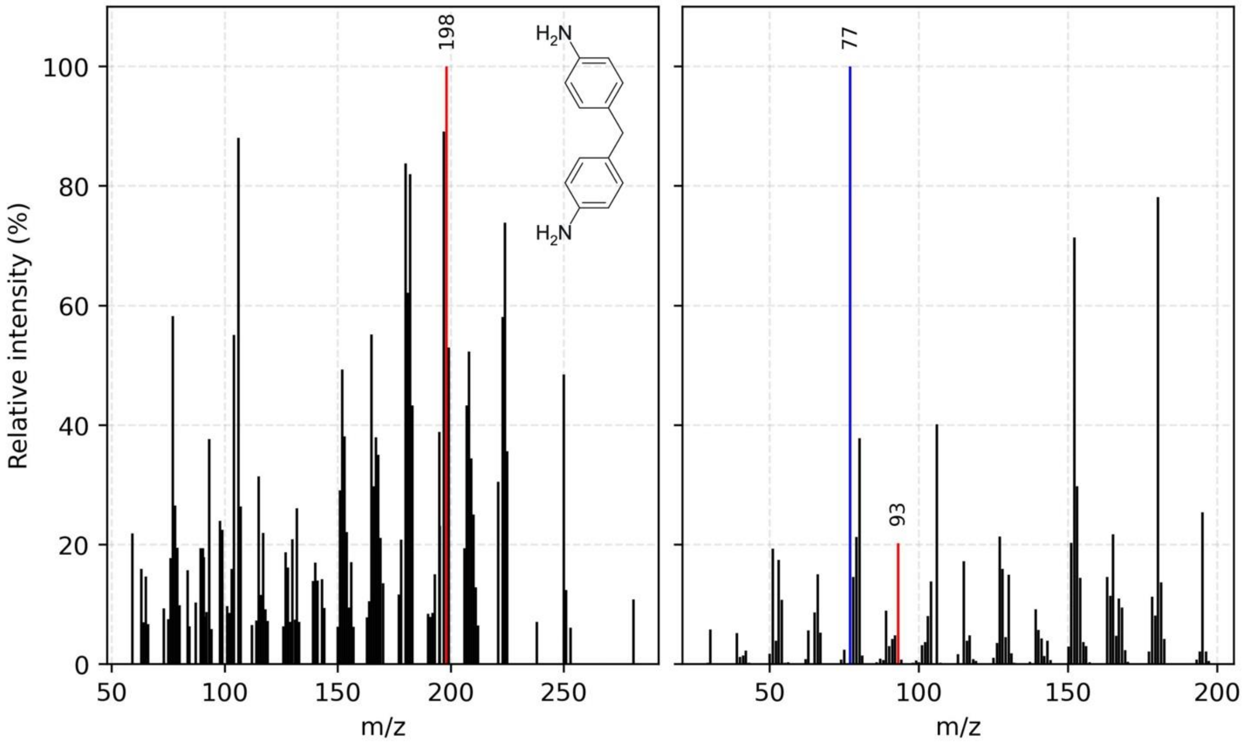

Figure S8. Full-scan (left) and product-ion (right) spectra for PUR acquired under optimized MS/MS conditions. Quantifier (red) and qualifier (blue) ions indicate the transitions used for quantitative analysis and identity confirmation.

Section 9. PS Mass Spectra

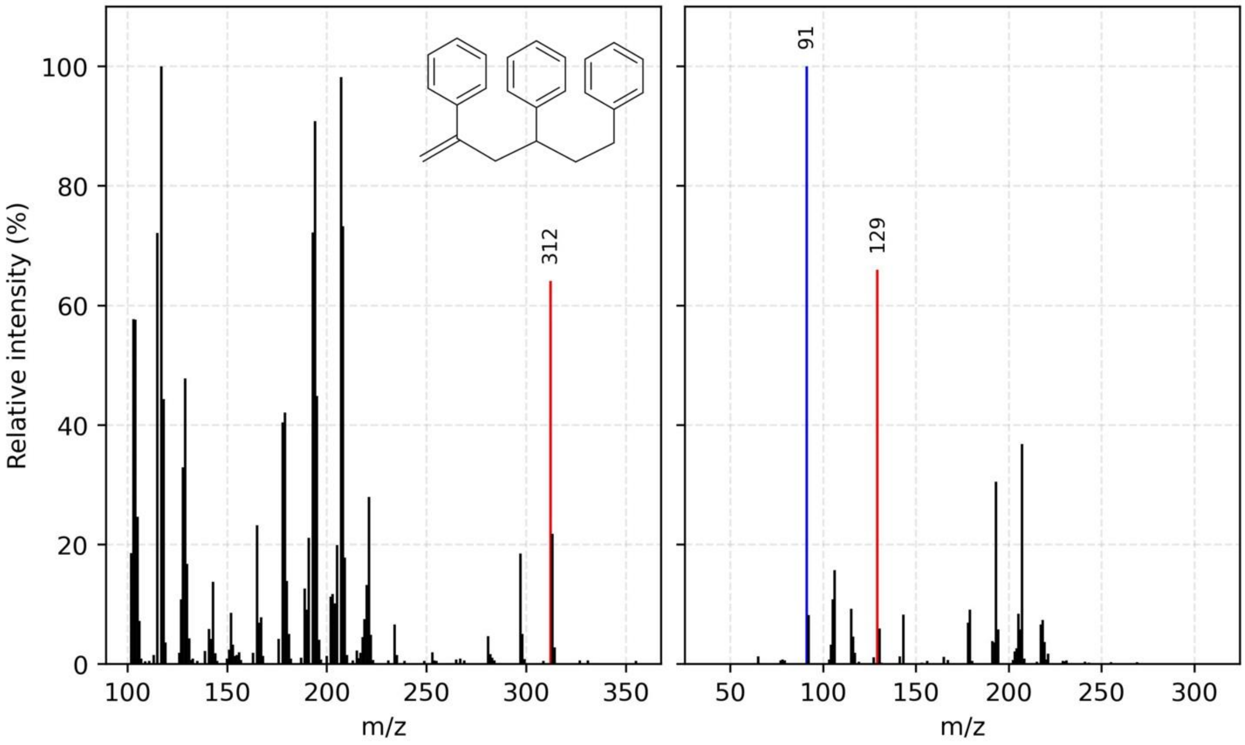

Figure S9. Full-scan (left) and product-ion (right) spectra for PS acquired under optimized MS/MS conditions. Quantifier (red) and qualifier (blue) ions indicate the transitions used for quantitative analysis and identity confirmation.

Section 10. PE Mass Spectra

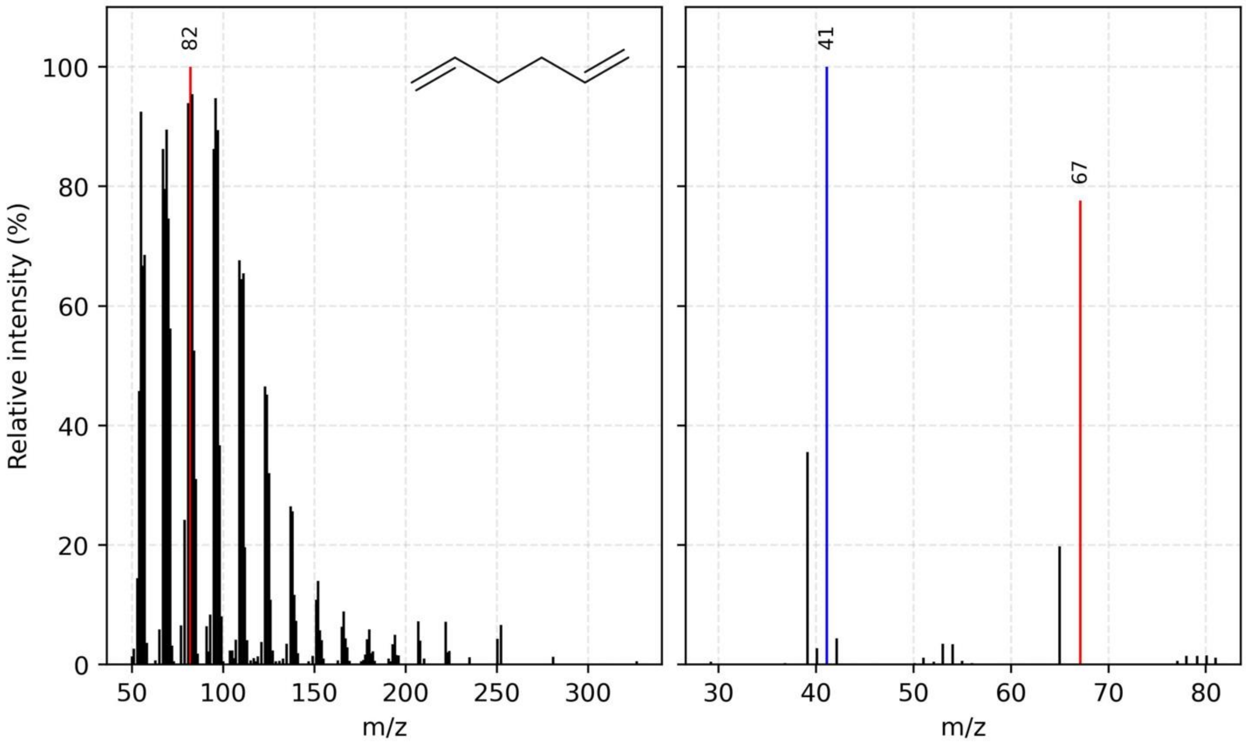

Figure S10. Full-scan (left) and product-ion (right) spectra for PE acquired under optimized MS/MS conditions. Quantifier (red) and qualifier (blue) ions indicate the transitions used for quantitative analysis and identity confirmation.

Section 11. PP Mass Spectra

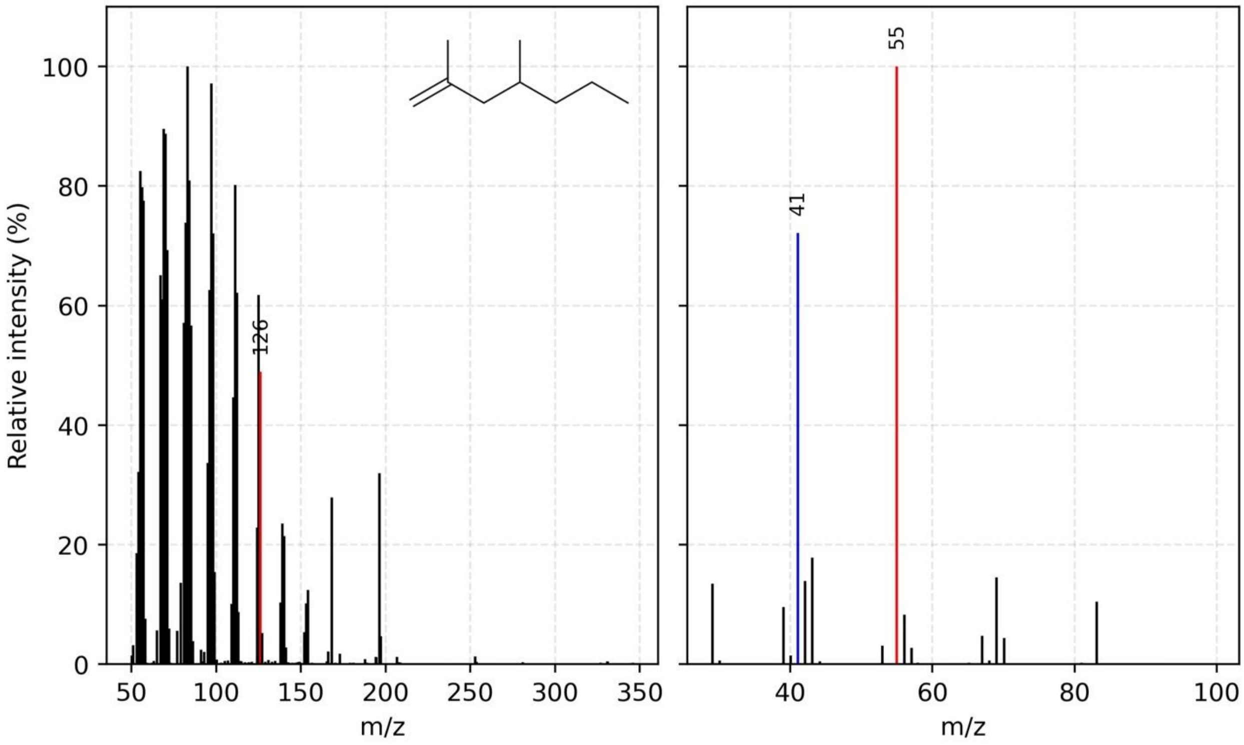

Figure S11. Full-scan (left) and product-ion (right) spectra for PP acquired under optimized MS/MS conditions. Quantifier (red) and qualifier (blue) ions indicate the transitions used for quantitative analysis and identity confirmation.

Section 12. ABS Mass Spectra

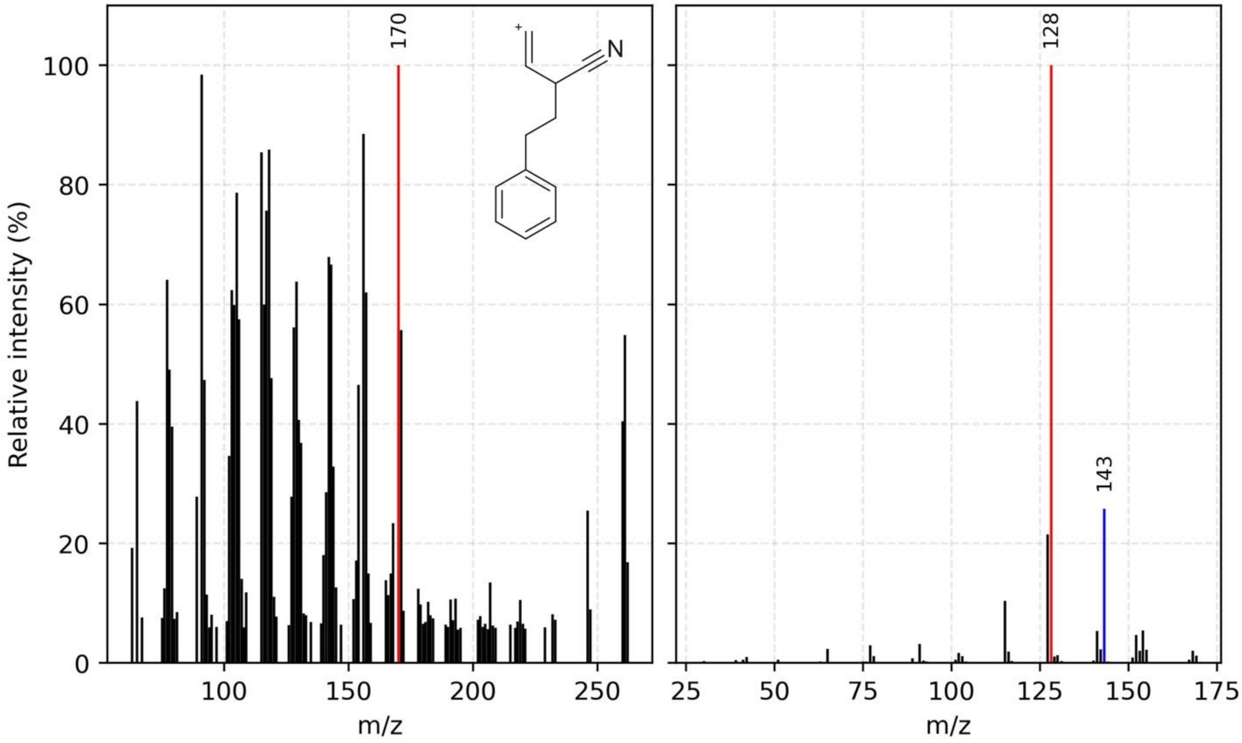

Figure S12. Full-scan (left) and product-ion (right) spectra for ABS acquired under optimized MS/MS conditions. Quantifier (red) and qualifier (blue) ions indicate the transitions used for quantitative analysis and identity confirmation.

Section 13. SBR Mass Spectra

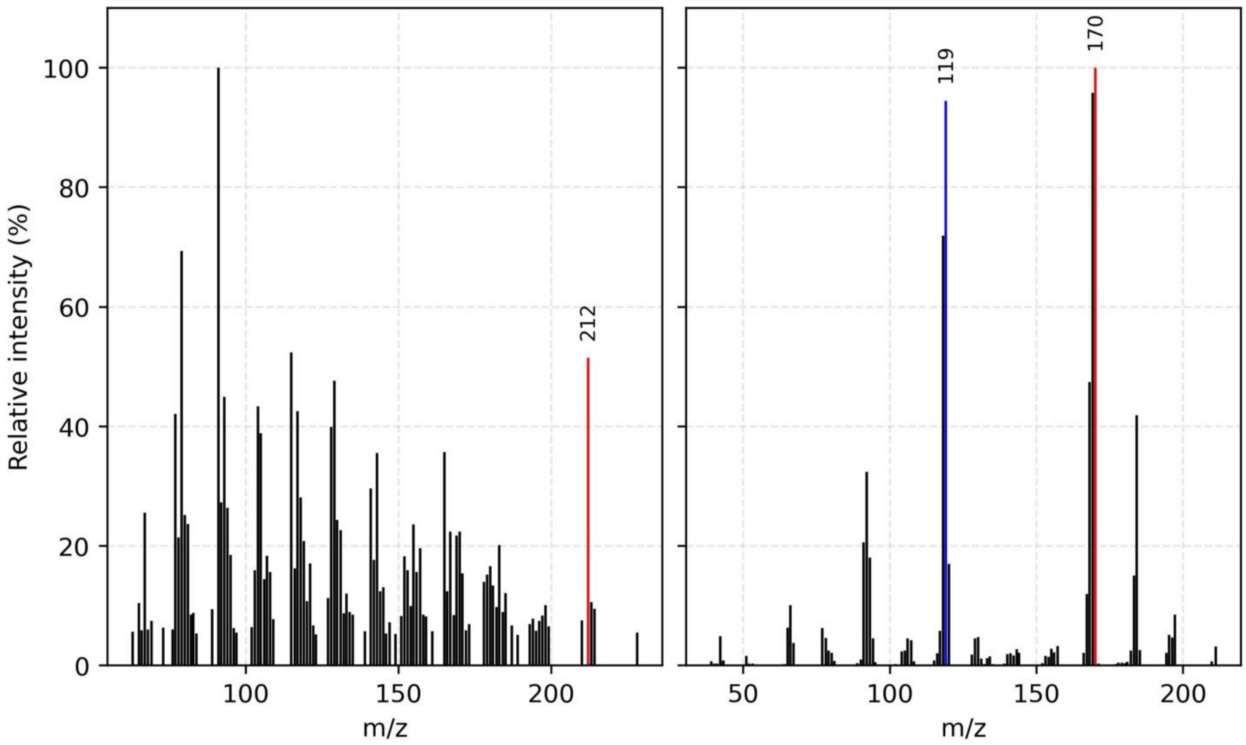

Figure S13. Full-scan (left) and product-ion (right) spectra for SBR acquired under optimized MS/MS conditions. Quantifier (red) and qualifier (blue) ions indicate the transitions used for quantitative analysis and identity confirmation.

Section 14. ASE Parameters

Table S1. ASE Parameters for Sediment and Lipid Extraction

Parameter	Sediment Extraction*	Lipid Extraction
Extraction solvent	Dichloromethane	50% Hexane; 50% Dichloromethane
Extraction temperature (°C)	180	100
Static time (s)	5	4
Cycles	3	2
Rinse volume (%)	80	40
Heating time (min)	9	5
Pressure (psi)	1500	1500
Purge time (s)	75	300
System rinse volume (mL)	9	9

*Parameters as described by Okoffo et al., 2023.

Section 15. GC-MS/MS Calibration Parameters

Table S2. Calibration Parameters and Quantifier/Qualifier ratios for 5 Point Calibrations.

Plastic	R^2	Max % Residuals	Quant:Qual
N-66	.9999	*0.5*	23
PE	.9999	2.0	84
PP	.9998	2.5	86
PVC	.9999	2.1	20
ABS	.9999	1.9	35
PA	.9999	2.8	101
PC	.9994	5.8	62
PET	.9997	8.5	52
PMMA	.9996	9.3	12
PS	.9995	4.1	65
PUR	.9995	3.6	6
SBR	.9999	2.8	99

Section 16. Lipid Correction Equations

Table S3. Lipid Correction Curve Equations. Where ‘y’ is the concentration to be corrected for and ‘x’ is the weight (mg) of lipid in each sample.

Plastic type	Correction Equation
Polymethylmethacrylate	$y = 14.663\ln(x) + 53.122$
Polypropylene	$y = 19.318\ln(x) + 65.210$
Polyvinyl chloride	$y = 21.347(x) + 2.327$
Polyamide	$y = 0.0676(x) + 0.1621$
Polycarbonate	$y = 0.0446\ln(x) + 0.2208$
Nylon-66	$y = 20.566(x) + 9.6908$
Polyethylene	$y = 33.197(x) + 50.514$
Polyethylene terephthalate	$y = 0.4316(x) + 2.0596$
Polyurethane	$y = -7E-05\ln(x) + 0.0804$
Acrylonitrile butadiene styrene	$y = 0.0012\ln(x) + 0.2025$
Styrene butadiene rubber	$y = 0.2707\ln(x) + 0.6483$
Polystyrene	$y = 3.312\ln(x) + 28.573$
